# Mitochondrial Electron Transport Chain in Heavy Metal-Induced Neurotoxicity: Effects of Cadmium, Mercury, and Copper

**DOI:** 10.1100/2012/136063

**Published:** 2012-04-24

**Authors:** Elena A. Belyaeva, Tatyana V. Sokolova, Larisa V. Emelyanova, Irina O. Zakharova

**Affiliations:** I. M. Sechenov Institute of Evolutionary Physiology and Biochemistry of Russian Academy of Sciences, Thorez pr. 44, 194223 Saint-Petersburg, Russia

## Abstract

To clarify the role of mitochondrial electron transport chain (mtETC) in heavy-metal-induced neurotoxicity, we studied action of Cd^2+^, Hg^2+^, and Cu^2+^ on cell viability, intracellular reactive oxygen species formation, respiratory function, and mitochondrial membrane potential of rat cell line PC12. As found, the metals produced, although in a different way, dose- and time-dependent changes of all these parameters. Importantly, Cd^2+^ beginning from 10 [mu]M and already at short incubation time (3 h) significantly inhibited the FCCP-uncoupled cell respiration; besides, practically the complete inhibition of the respiration was reached after 3 h incubation with 50 [mu]M Hg^2+^ or 500 [mu]M Cd^2+^, whereas even after 48 h exposure with 500 [mu]M Cu^2+^, only a 50% inhibition of the respiration occurred. Against the Cd^2+^-induced cell injury, not only different antioxidants and mitochondrial permeability transition pore inhibitors were protective but also such mtETC effectors as FCCP and stigmatellin (complex III inhibitor). However, all mtETC effectors used did not protect against the Hg^2+^- or Cu^2+^-induced cell damage. Notably, stigmatellin was shown to be one of the strongest protectors against the Cd^2+^-induced cell damage, producing a 15–20% increase in the cell viability. The mechanisms of the mtETC involvement in the heavy-metal-induced mitochondrial membrane permeabilization and cell death are discussed.

## 1. Introduction

The highly toxic heavy metals, such as cadmium (Cd^2+^), mercury (Hg^2+^), and copper (Cu^2+^), are environmentally and occupationally widespread pollutants with mutagenic, carcinogenic, and teratogenic effects [1–4]. Being either nonessential (Cd, Hg) or biogenic (Cu) elements, these metals belong to the most harmful factors due to their tendency to accumulate in tissues and organs and to transfer along food chains, high reactivity (in particular, high affinity to thiol groups), and the ability to stimulate reactive oxygen species (ROS) formation and to produce injury in cell functions. In addition, a characteristic feature of Cd^2+^ is its ability to act not only as a dithiol reagent but also as a Ca^2+^ agonist most probably due to the extreme closeness of crystal ionic radii of the ions (Cd^2+^—0.097 nm; Ca^2+^—0.099 nm) [5].

Although it is well known now that mitochondria are important targets for heavy metals; nevertheless, mechanism(s) of the disturbance of mitochondrial function by heavy metals are not well understood. Not long ago, on rat hepatoma AS-30D cells we have demonstrated that the intracellular ROS changes and mitochondrial dysfunction are involved in cytotoxicity mechanism(s) of such heavy metals as Cd^2+^, Hg^2+^, and Cu^2+^ [6, 7]. Moreover, we have found [6] that Cd^2+^-induced AS-30D cell death is accompanied by stimulation of ROS production at the mitochondrial respiratory chain complex III level and opening of the mitochondrial permeability transition (MPT) pore (for reviews see [8–11]).

The MPT pore can be defined as a voltage-dependent, nonselective high-conductance inner mitochondrial membrane channel of unknown molecular structure, which allows solutes up to 1500 Da to pass freely in and out of mitochondria. The MPT pore opens under conditions of calcium overload; the opening is greatly enhanced by adenine nucleotide depletion, elevated phosphate, and oxidative stress. The opening of the MPT pore *in vivo* produces ATP pool exhaustion, disturbance of Ca^2+^ homeostasis, and efflux of various apoptotic factors from mitochondria [8, 9]. As accepted by the most, the MPT pore represents by itself a transmembrane multiprotein complex located at contact sites between two mitochondrial membranes; the minimum MPT pore configuration likely consists of the matrix protein cyclophilin-D (CyP-D), the potent inhibitor of which is cyclosporin A (CsA), of the adenine nucleotide translocase (ANT) and/or phosphate carrier (P_i_C) (inner membrane proteins), and the voltage-dependent anion channel or porin (outer membrane protein); however, the involvement of the latter is under doubts at the moment [10, 11]. As considered previously, the ANT represents a crucial core element of the MPT pore; however, the latest evidence indicates that the ANT usually plays a regulatory role rather than provides the transmembrane pore component and point to the P_i_C as the protein, fulfilling the latter role. The data obtained by Halestrap's group during the last years are consistent with a model of the MPT pore, in which a calcium-triggered conformational change of the P_i_C, facilitated by CyP-D, induces the pore opening that is enhanced by an association of the P_i_C with the “c” conformation of the ANT. An interaction of the P_i_C with the ANT may enable agents that bind to either transporter to modulate the pore opening [10]. The MPT pore opening is inhibited by CsA and different ANT inhibitors, in particular, bongkrekic acid (BKA). The pore opening is strongly promoted by the oxidized state of pyridine nucleotides (PNs) and of critical dithiols, at least, at two discrete redox-sensitive sites, P- and S-site(s), respectively; their localization is still unknown; however, the existence of critical internal and external dithiols is suspected [9, 11, 12]. The MPT pore is characterized by pH- and voltage-dependence, namely, it is closed at acidic pH (intracellular pH below 7.0) and at high transmembrane potential values. The MPT pore function is modulated by electron flux via respiratory complex I, by different ubiquinone analogs, and various regulatory proteins [9]. As known, Ca^2+^ is a critical factor for opening of the “classical” CsA-sensitive MPT pores. The most of other bivalent metal ions behave as the pore inhibitors, and this is true irrespective of whether cations are accumulated (like Sr^2+^, Mn^2+^, and Ba^2+^) or not (like Mg^2+^). The experimental data point to the existence of two separate Me^2+^-binding sites on the MPT pore complex-external (inhibitory) and internal (activating); however, their molecular localization is uncertain up-to-date. H^+^ is considered to compete with Ca^2+^ on the Ca^2+^-trigger site as well, and histidine nature of this site is suspected [8–12]. There are some interesting suppositions in literature in the field about the Me^2+^-binding sites localization. In particular, it was proposed that respiratory complex I [9], a cardiolipin core of the ANT [13], or its matrix loops [14], could be the hypothetical places of their localization. We also suggested that the respiratory complex III may contain Me^2+^(Cd^2+^)-binding loci, crucial for the MPT pore modulation [15]. Further, we hypothesized about possible involvement of the mitochondrial respiratory chain supercomplex, formed by complex I (P-site) and complex III (S-site), in the mitochondrial membrane permeabilization mediated by the MPT pore ([16–18], for details, see Section 4).

The present study was conducted to underscore the role of mitochondria and mitochondrial electron transport chain (mtETC) in heavy metal-induced neurotoxicity, using neuron-like rat PC12 cells and the divalent heavy metal ions as a model system. Portions of this investigation were presented before in an abstract form and as a part of a published lecture [19–21].

## 2. Materials and Methods

### 2.1. Chemicals

The most of reagents was purchased from Sigma Aldrich Company (St. Luis, MO, USA). Cyclosporin A was from Novartis (Basel, Switzerland). The rest of the chemicals was of the highest purity, commercially available. The DMEM incubation medium (Dulbecco/Vogt modified Eagle's minimal essential medium) with L-glutamine, horse blood serum, fetal calf serum, and trypsin-EDTA were purchased in Biolot Company (Russia).

### 2.2. Cell Culture

The experiments were made on cultures of PC12 cells in CO_2_ incubator in the atmosphere containing 5% of CO_2_ at 37°C as before [22]. As the incubation medium DMEM with L-glutamine was used, containing 25 *μ*g/mL of penicillin and 25 U/mL of streptomycin, 5% of horse blood serum and 10% of fetal calf serum. The incubation medium was changed every 2-3 days. In some experiments, the incubation medium was DMEM (with L-glutamine and antibiotics) not containing serum. PC12 cells were seeded to 24 well plates in concentration 2.5 × 10^5^ cells in each well to make assays of lactate dehydrogenase (LDH) release or TMRM fluorescence (see below). The experiments started 24 h after the transfer of the cells to the plates or Petri dishes. The preincubation of PC12 cells with different effectors or without them was performed for 30 min at 37°C prior to the exposure of the cells to the heavy metals. Then, to each well or Petri dish, the corresponding concentration of Cd^2+^, Hg^2+^ or Cu^2+^ was added, in particular 10, 50, 100, or 500 *μ*M. CdCl_2_, HgCl_2_, or CuCl_2_ was dissolved in water (10 mM stock solution).

### 2.3. Cell Viability Assay

The viability of the cells and protective action of different antioxidants, inhibitors of the mitochondrial permeability transition, and the respiratory chain against the heavy-metal-induced cytotoxicity was estimated by spectrophotometric monitoring of the LDH cellular release [22]. The samples were centrifuged for 5 min at 200–300 g to pellet down cells and supernatants collected. The lysis of the cells was performed by using 1% Triton X-100 at room temperature, and then the total activity of LDH was determined in the samples. In particular, the LDH activity was determined measuring NADH level for this purpose. The decrease in optical density of the samples at 340 nm was registered during 5-6 min as previously described [23], using the spectrophotometer M40 (Karl-Zeisse, Germany). The reaction was performed in the medium composed of (mM): 80 Tris-HCl (pH 7.2), 200 NaCl, 1.6 pyruvate, 0.2 NADH. The percent of LDH activity released was determined as the percent of enzyme activity in the incubation medium to the total LDH activity in the sample. The absence of the viable cells corresponds to 100% of LDH activity in the incubation medium. The cell viability was expressed in % of the untreated control.

### 2.4. Cell Respiration Assay

The cell respiration was determined polarographically with the help of Clark oxygen electrode in a thermostatic water-jacketed vessel with magnetic stirring at 37°C. PC12 cells (10^7^ cells) were incubated in 10 mL of the complete DMEM medium (with serum) in Petri dishes for 3, 5, 24, and 48 h with various concentrations of the corresponding heavy metal, then collected by centrifugation and transferred to the DMEM medium without serum. Under these conditions, oxygen uptake by the cells was used to sustain a steady-state level of mitochondrial membrane potential (ΔΨ_mito_) by compensating for the proton leak and ATP synthesis by mitochondrial ATP synthase (F_0_F_1_-ATPase). This was designated as the “steady-state” or basal respiration. Addition of the inhibitor of F_0_F_1_-ATPase, oligomycin, typically decreased cell respiration, leaving only that portion of oxygen uptake, which compensated for the proton leak (“resting respiration”, state 4o). A subsequent addition of the chemical protonophore FCCP (carbonyl cyanide *p*-trifluoromethoxyphenylhydrazone) resulted in the maximal rate of respiration, limited only by capacity of the respiratory chain (“uncoupled respiration”, state 3u) [24]. Such protocol enables us to follow the effect of poisoning by heavy metal ions on distinct steps of the mitochondrial energy-coupling process and to estimate cell respiratory control ratios (RCRs) and some additional parameters (coupling efficiency, spare respiratory capacity) that are rich in information about mitochondrial function and dysfunction in intact cell [25]. 

### 2.5. ROS Production Assay

The intracellular ROS production was measured spectrofluorometrically using the fluorescent dye dichlorodihydrofluorescein diacetate (DCFH_2_-DA) as the ROS-sensitive probe [26, 27]. PC12 cells were seeded to 6 well plates to determine ROS formation and accumulation (1 × 10^6^ of cells in a well). The experiments started 24 h after the transfer of the cells to the plates, they were performed in DMEM containing L-glutamine and this medium will be described as DMEM. The incubation with inhibitors and heavy metals was made as previously described. 20 min before the end of each incubation, DCFH_2_-DA was added to the incubation medium in final concentration of 10 *μ*M. In order to get rid of the dye excess, the cells were washed by Hanks' balanced salt solution. The fluorescence of the reaction product of ROS with dichlorodihydrofluorescein was estimated at the spectrofluoremeter Schimadzu 1501 (Japan), measuring the emission at *λ* = 522 nm, the length of the excitation wave being equal to 475 nm [26]. The ROS content was measured in arbitrary units reflecting the intensity of the fluorescence of reaction product. In some cases, DCF fluorescence was measured also by Fluoroscan FL (ThermoFisher) using Em = 538 nm, Ex = 485 nm.

### 2.6. ΔΨ_mito_ Changes Assay

ΔΨ_mito_ was determined with the aid of a cationic fluorescent probe, TMRM (tetramethylrhodamine methyl ester) [28]. All incubations were conducted in the DMEM medium as described before (see above). Then, PC12 cells were suspended in Hanks' balanced salt solution (10^6^ of cells in mL) and were loaded with TMRM (200 nM) for 30 min at 37°C in the dark. Afterwards, the cells were centrifuged for 3 min at 60 g and resuspended in PBS (phosphate buffered saline) medium. The samples were analyzed by flow cytometry. A flow cytometer EPIXC-XL (Beckman Coulter, USA) was used equipped with an argon laser emitting at 488 nm. Orange fluorescence caused by TMRM was detected in FL2 575 ± 15 nm. Approximately, 20,000 events per sample were acquired for the analysis performed. The fluorescence intensity of TMRM (reflecting ΔΨ_mito_) was monitored on a log scale and expressed as mean fluorescence intensity, MnI (in arbitrary units). The flow cytometric data were analyzed with WinMDI 2.9 (Joseph Trotter, Scripps Research Institute, La Jolla, CA, USA). The fluorescence of samples in the presence of inhibitors is expressed as the percentage of controls (without inhibitors).

### 2.7. Statistics

Data are expressed as mean values ± SE for at least three independent experiments, unless otherwise indicated. The statistics were analyzed with ANOVA and Student's *t*-tests, with *P* < 0.05 assumed as the significance threshold.

## 3. Results

To understand molecular mechanism(s) of heavy-metal-induced cell dysfunction and the role of mitochondria in neurotoxic action of such heavy metals, as Cd^2+^, Hg^2+^, and Cu^2+^, we investigated the effects of these divalent metal ions on cell viability, intracellular ROS formation, ΔΨ_mito_, and respiratory function of rat neuron-like PC12 cells. The cell viability was estimated by LDH release from the cells, the respiration rates were measured polarographically, the ROS production—spectrofluorometrically using DCFH_2_-DA, and the membrane potential—with the help of flow cytometry and a fluorescent probe TMRM.

**Figure 1:**
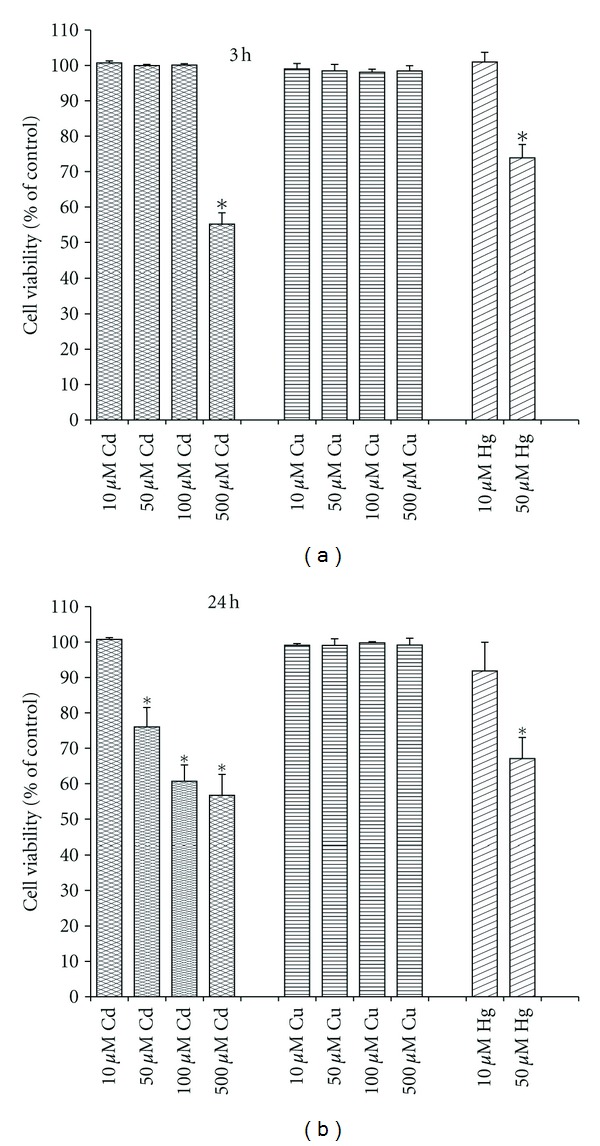
Time- and dose-dependent response to Cd^2+^, Cu^2+^, and Hg^2+^ on PC12 cell viability assessed by LDH release. The results are expressed in % to corresponding control (*P < 0.05 compared to untreated control; *n* = 4–9 independent experiments).

We found that already after 3 h of incubation of the cells with 50 *μ*M Hg^2+^, the cell viability decreased on 25%; the same [Cd^2+^] and its twofold increase were not effective, whereas 500 *μ*M Cd^2+^ produced close to the 50% decline of the cell viability at that time (Figure 1). 100 *μ*M Cd^2+^ reduced the cell viability on 25% only after 5 h treatment (Tables 4 and 5). It is important to say that the highest [Cu^2+^] used (i.e., 500 *μ*M) became effective only after 48 h of incubation of the cells with the metal; however, even in this case, the cell viability reduced not more than 10% (not shown data).

**Figure 2:**
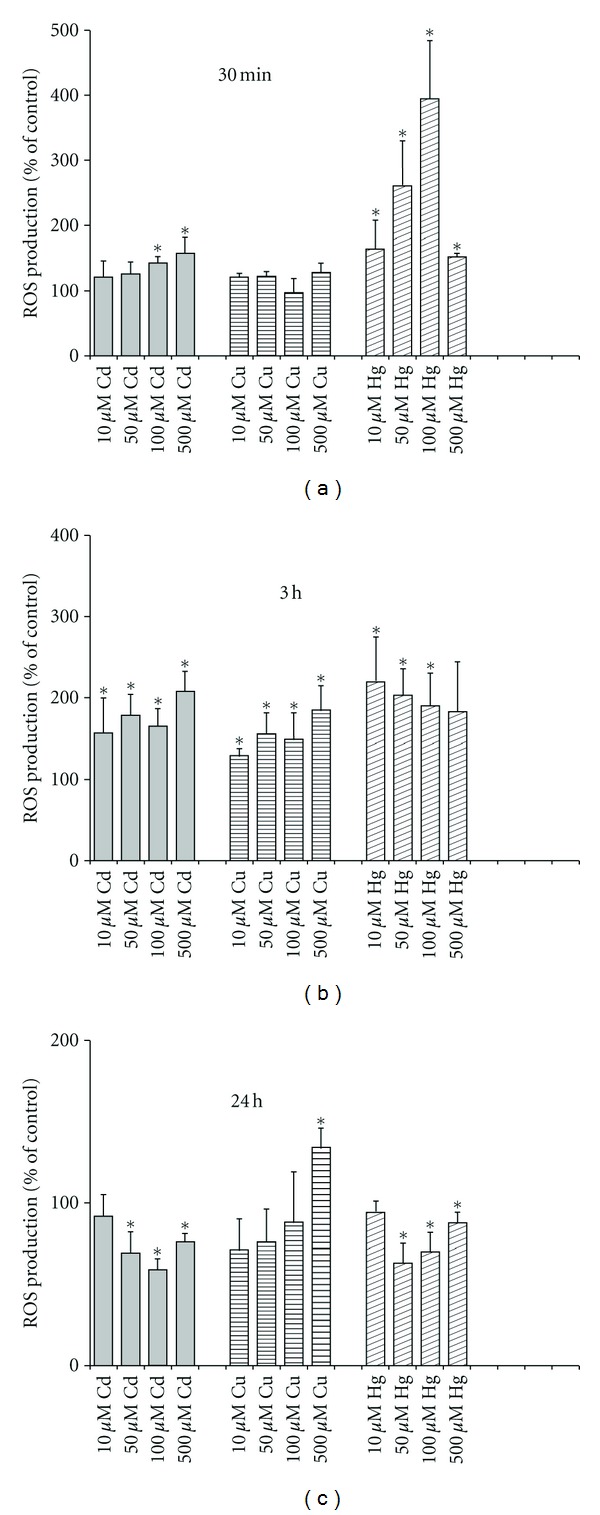
Time- and dose-dependent effects of Cd^2+^, Cu^2+^, and Hg^2+^ on ROS formation by PC12 cells measured spectrofluorometrically using DCFH_2_-DA as the ROS-sensitive probe. The results are expressed in % to corresponding control (*P < 0.05 compared to untreated control; *n* = 3–7 independent experiments).

All heavy metal ions under study produced, although in a different way, dose- and time-dependent changes in intracellular ROS generation of PC12 cells. In particular, 100 and 500 *μ*M of Cd^2+^ induced a significant increase in the ROS formation after 30 min of incubation with the cells, whereas after 3 h already all Cd^2+^ concentrations under test stimulated strongly the intracellular ROS production (Figure 2). It is seen also that after 24 h of incubation of the cells with the metal, the ROS level in the presence of 10 *μ*M Cd^2+^ returned to baseline. At the same time, 50–500 *μ*M Cd^2+^ decreased significantly the ROS production compared to control level. As to Hg^2+^, already during short incubation time (30 min) all concentrations of the metal studied enhanced the intracellular ROS generation (Figure 2). Moreover, again, like in the case of Cd^2+^, after 24 h incubation of the cells in the presence of 10 *μ*M of Hg^2+^, the ROS production level reached the baseline whereas 50–500 *μ*M Hg^2+^ significantly decreased the ROS production compared with the control. In the case of Cu^2+^, there were no significant changes in the ROS production after 30 min of incubation while already after 3 h all Cu^2+^ concentrations under test induced the significant stimulation of the ROS formation (Figure 2). Notably, the ROS generation level observed in the presence of 500 *μ*M Cu^2+^ was high even after 24 h of incubation of the cells with the metal. 

**Figure 3:**
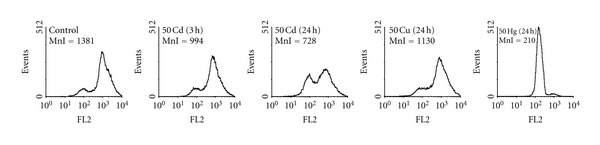
Effects of Cd^2+^, Cu^2+^, and Hg^2+^ on mitochondrial membrane potential of PC12 cells monitored by flow cytometry after staining the cells with the lipophilic cationic probe TMRM. MnI—mean intensity of fluorescence (arbitrary units) is indicated in the upper left corner of each panel. A typical experiment out of at least three independent ones for each metal is shown. For other details, see Section 2.

To underscore the involvement of mitochondria in the harmful effects of the metals, we studied their influence on ΔΨ_mito_ of PC12 cells. As seen from Figure 3, 50 *μ*M Cd^2+^ reduced the ΔΨ_mito_ more than 25% after 3 h of exposure with the cells, and after 24 h of treatment the ΔΨ_mito_ loss reached to 50%. Notably, during 24 h of treatment, 50 *μ*M Cu^2+^ did not produce any significant action on the ΔΨ_mito_ whereas 50 *μ*M Hg^2+^ evoked practically the complete ΔΨ_mito_ dissipation at this time (Figure 3). 

For assessing mitochondrial dysfunction in cells (see [25]) and the underlying mechanisms, we examined action of the metals on respiration of PC12 cells. With this purpose, we measured the rates of the basal respiration (Table 1), of the resting respiration (Table 2), and of the uncoupled respiration (Table 3) in the absence and in the presence of different concentrations of the heavy metals and after different time of exposure, namely, 3, 5, 24, and 48 h.

It should be remind that the participation of the mtETC could be checked by estimation of the uncoupled respiration rate, the inhibition of which is considered a marker of the disturbance of the respiratory chain components. Cd^2+^, beginning from 10 *μ*M and already at short incubation time (3 h), produced significant inhibition of FCCP-uncoupled respiration of the cells (Table 3). The complete inhibition of the mtETC was reached after 3 h of treatment with 50 *μ*M Hg^2+^ or 500 *μ*M Cd^2+^ and after 24 h of treatment with 100 *μ*M Cd^2+^, whereas even after 48 h of incubation of the cells with 500 *μ*M Cu^2+^, only 50% inhibition of the maximum respiration rate took place. In opposite, the restoration of the uncoupled respiration rate up to the control level occurred in the presence of 10 *μ*M Cd^2+^ after 24 h of incubation or in the presence of 10 *μ*M Hg^2+^ after 48 h of incubation (Table 3). As to Cu^2+^, only at high concentrations (100 and 500 *μ*M) and starting from 24 h of treatment, the significant changes in the cellular respiration occurred (Tables 1–3). Notably, at 500 *μ*M Cu^2+^ and after 48 h of incubation, all three values (i.e., the steady-state respiration, the resting respiration, and the uncoupled respiration) were strongly depressed, indicating a potent inhibitory effect on the respiratory chain. 

It is known also that the cellular respiratory rate in the presence of oligomycin (the resting respiration rate) is a direct measure of the proton leak across the mitochondrial membrane *in situ* [25]. As seen from Table 2, after 48 h incubation of the cells with 10 *μ*M Hg^2+^, the resting respiration was stimulated significantly, whereas the uncoupled respiration remained unaffected (Table 3), pointing to some uncoupling effect of that low concentration of the metal. At the same time, the basal respiration in the presence of 10 *μ*M Hg^2+^ was significantly enhanced as well (Table 1). So, after simple calculations, it becomes evident that low Hg^2+^ substantially reduced the coupling efficiency of the cells whilst did not affect the spare respiratory capacity of the cells (see Section 2 and [25]).

Further, we studied the action of different effectors of the respiratory chain on the viability of PC12 cells in the presence of the heavy metals which was determined by the LDH release from the cells as before (Table 4). We used two concentrations (0.1 and 1 *μ*M) of FCCP (an artificial uncoupler) and of several selective inhibitors of the mtETC components, namely, of complex I (rotenone) and complex III (myxothiazol, antimycin A, and stigmatellin). Among the mtETC effectors under test, only stigmatellin (1 *μ*M) produced the strong and sustained neuroprotection, increasing the cell viability on 15–20% (Table 4). In the case of Hg^2+^ (50 *μ*M; 3 h) and Cu^2+^ (500 *μ*M; 48 h), all mtETC effectors under study did not exhibit significant protective action on the cell survival (data not shown). All this means that the mtETC dysfunction is involved (however, in a different way) in mechanism(s) of neurotoxic action of the heavy metals.

Finally, we investigated action of different antioxidants and inhibitors of the MPT pore on the Cd^2+^-induced ROS-dependent injury of PC12 cells. We found that all antioxidants under study, namely, N-acetylcysteine (NAC), vitamin E (vit E), butylhydroxytoluene (BHT), 2,2,6,6-tetramethylpiperidine-1-oxyl (TEMPO), and mannitol were effective, however, in a different extent, in protection of the cells against the Cd^2+^ toxic action, with maximal neuroprotective effect observed after 2 h preincubation of the cells with 10 mM NAC (Table 5). The latter treatment was found to be highly protective against the Hg^2+^-induced cytotoxicity as well (not shown data). Besides, we revealed that Ruthenium Red, RR, or Ru-360 (selective inhibitors of mitochondrial Ca^2+^ uniporter, MCU) both taken in concentration of 10 *μ*M were protective only at short times of incubation of the cells with Cd^2+^ (3 h—for 500 *μ*M Cd^2+^ and 5 h—for 100 *μ*M Cd^2+^), increasing the cell viability on 16% and 18%, respectively. By 24 h, both 10 *μ*M and 50 *μ*M of RR did not protect against the 100 *μ*M Cd^2+^-induced cell death. As seen from Table 5, CsA (a potent pharmacological inhibitor of the MPT pore) also produced only the transient protection against the Cd^2+^-induced cytotoxicity; in particular, after 3 h, the cell viability in the presence of CsA (1 *μ*M) rose about 20%, whereas at the longer incubations of the cells with Cd^2+^, it lost the protective action. The same was true for another MPT pore inhibitor—BKA (selective inhibitor of ANT). Both 5 *μ*M and 25 *μ*M of BKA were effective only after 3 h incubation of the cells with Cd^2+^, increasing the viability approximately on 15%. It is necessary to say that all used concentrations of the effectors under test *per se* were not cytotoxic under used conditions.

We obtained also that Ru-360 (10 *μ*M), vit E (500 *μ*M), FCCP (1 *μ*M) did not change significantly the ROS formation increase induced by 100 *μ*M Cd^2+^ after 30 min incubation with the cells, whereas CsA (1 *μ*M) and BKA (7 *μ*M) significantly decreased the ROS production in the presence of Cd^2+^. However, in the case of 3 h of incubation with Cd^2+^, BKA lost its protective effect, while vit E, in opposite, became effective. As to FCCP, after 3 h exposure of the cells with 100 *μ*M Cd^2+^, this protonophoric uncoupler reduced in an equal degree (about 30%) both the ROS production of the Cd^2+^-treated and the control cells. In turn, stigmatellin (1 *μ*M) did not affect significantly the ROS generation stimulated by 30 min incubation of the cells with 100 *μ*M Cd^2+^, whereas it reduced on 12% the ROS production enhanced by 3 h incubation of the cells with the same [Cd^2+^]. This small but significant effect of stigmatellin was observed despite that this concentration of the complex III inhibitor *per se* stimulated the ROS production of the control cells, upon the average, on 25%.

## 4. Discussion

The work was made with the aim to elucidate the role of mitochondrial respiratory chain in the heavy-metal-induced neurotoxicity. We studied action of Cd^2+^, Hg^2+^, and Cu^2+^ on cell viability (Figure 1; Tables 4 and 5), respiratory function (Tables 1–3), intracellular ROS generation (Figure 2), and ΔΨ_mito_ (Figure 3) of rat neuron-like PC12 cells. As found, the metals produced, however, differently, dose- and time-dependent changes in all these parameters, with Hg^2+^ being the most neurotoxic.

It is known that the measurement of cell respiratory control is the single most useful general test of mitochondrial function in cell populations [25]. The ratio of the uncoupled rate (state 3u) to the rate with oligomycin present (state 4o) is analogues to the (uncoupled) respiratory control ratio of isolated mitochondria. It is sensitive to changes in substrate oxidation and proton leak (not to ATP turnover) and is a reliable marker of mitochondrial dysfunctions in cells. Using data from Tables 2 and 3, it is possible to estimate the apparent respiratory control ratios of the cells (state 3u/state 4o) in the presence of the heavy metals and to see the dynamics of their changes. As evident, the RCR changed crucially after treatment of the cells with the heavy metals. In addition, under used conditions, the dissipation of ΔΨ_mito_ was observed in the presence of Cd^2+^ and Hg^2+^ (Figure 3). All this means that the mitochondrial dysfunction participates in mechanisms of the cell dysfunction produced by the heavy metals. Besides, the data on the ROS production (Figure 2) and the cell viability (Figure 1) confirmed our previous suggestion [7] that the increased ROS level alone was not sufficient to induce the cell death by heavy metals and additional factor(s) must have been present that was/were responsible for their cytotoxic action, most likely the blockage of the respiratory chain (see Table 3). The results found in this study are in a good accordance with data shown before on PC12 cells [29–40] and on other types of cells, which obtained by us and other authors ([41–47]; see also [6, 7] and references therein). 

As known, the ability of Cd^2+^ to act not only as SH reagent but also as a Ca^2+^ agonist makes it an excellent tool to study the MPT, with the aim to solve the problem of the proposed direct participation of the respiratory chain components in this phenomenon and in cell death regulation ([15] and references therein). We discovered herein that the severe mitochondrial dysfunction manifested in the mtETC disturbance and the MPT pore opening was critically involved in mechanism of neurotoxic action of Cd^2+^. Moreover, on the basis of the obtained in this work evidence, it is possible to conclude that thiol status and the ROS generation changes are important participants of the events which contribute in the cell death induced by Cd^2+^. In particular, we found that NAC (GSH precursor), RR (MCU inhibitor), CsA and BKA (MPT pore inhibitors), FCCP (protonophoric uncoupler), stigmatellin (complex III inhibitor), and different antioxidants partially prevented the Cd^2+^-induced necrosis (Tables 4 and 5). Notably, in Cd^2+^-treated cells, the dose- and time-dependent increase of intracellular ROS production was partially depressed by CsA, BKA, vit E, and stigmatellin. In contrast, FCCP reduced in an equal extent both the ROS production of the Cd^2+^-treated and of the control cells. All this means that the increase in the ROS generation by Cd^2+^ is one of the important events in the heavy-metal-produced cytotoxicity as well as underscores the mitochondrial origin of the phenomenon. Moreover, it is likely directly connected with complex III of the mtETC, in particular with its Q_o_ site. Besides, the differences in the effects of stigmatellin and myxothiazol (Table 4) indicate that the increase of ROS formation likely involves the mobility of iron-sulfur protein (ISP) of bc_1_ complex (i.e., complex III) as a key feature that is in line with previous observations of Armstrong et al. [48] and Muller et al. [50]. It is important that the MPT pore opening is likely also involved in this process that is in a good accordance with findings of He and Lemasters who, using proteomics, revealed that after induction of the MPT, dephosphorylation of the Rieske ISP of complex III took place [51]. In addition, there is interesting evidence in literature in the field that in the binding to complex III of such heavy metal as Zn^2+^, which partially depressed by Cu^2+^, ethoxyformic anhydride (i.e., histidyl residues modifier), and *N-*ethylmaleimide, NEM, the Rieske ISP participates as well ([52], see also [15], and references therein). At last, the absence of significant action of rotenone (complex I inhibitor) confirms the critical role of complex III in the Cd^2+^-induced neurotoxicity (Table 4).

The results, revealed by us on the intact cells herein and before (PC12 and AS-30D [6, 7]), are reinforced by our previous observations on isolated rat liver mitochondria (RLM) [15–18, 49, 53] where we studied the action of different mtETC and MPT pore inhibitors on the heavy-metal-induced high-conductance mitochondrial swelling in isotonic sucrose medium (a marker for the MPT pore involvement in mitochondrial membrane alterations) [8]. We found that Cd^2+^-induced high-amplitude swelling in isotonic sucrose medium (highly sensitive to the combined action of CsA, ADP, Mg^2+^, and dithiothreitol, DTT, [15, 17, 53]) was also sensitive to BKA, rotenone, and stigmatellin [18, 49]. Furthermore, our data agree well with the results obtained by other workers on isolated mitochondria [54, 55]. Not long ago, Wang and coauthors showed that Cd^2+^ stimulated ROS production in isolated liver, brain, and heart guinea pig mitochondria; moreover, their observations indicated that complex III might be the only site of ROS production induced by Cd^2+^ [54]. Besides, the results of their kinetic studies and electron turnover experiments confirmed and extended the previous findings of Miccadei and Floridi on isolated RLM [55] and gave them possibility to suggest that Cd^2+^ might bind between semiubiquinone and cytochrome b_566_ of the Q_0_ site of complex III, resulting in accumulation of semiubiquinones at the Q_0_ site [54]. In addition, on isolated rat hepatocytes, there was shown that just mitochondria were ROS sites for such non-redox or poor redox cycling transition metals as Cd^2+^, Hg^2+^, and As^3+^ [56]. Recently, it was found also that cytotoxic action of Cd^2+^ was blocked not only by the mitochondrial antioxidant *α*-lipoic acid but also by the mitochondrial complex III bypass agent 2,6-dichloroindophenol, DCIP (i.e., selective complex III electron acceptor) [45].

It should be mentioned, however, that there is some cell type specificity in toxic action of Cd^2+^, which is known to induce both necrosis and/or apoptosis, and even can produce antiapoptotic effect [57, 58]. For example, in human hepatoma HepG2 cells [59], Cd^2+^ produced a rapid and transient ROS generation, mitochondrial dysfunction, and apoptosis; moreover, the Cd^2+^-triggered apoptosis was not inhibited by CsA and BKA while it was blocked by NAC pretreatment. In some other cells [42, 60, 61], the Cd^2+^-induced apoptosis was mitochondria- and ROS-dependent event that, in opposite, was inhibited by oligomycin [61], CsA [42, 61], and in all cases was depressed by rotenone and antioxidants. In this connection, it is worthy to remind that rotenone decreases ROS production by complex III while enhances ROS production by complex I and the relative contribution of the two complexes to ROS production may vary in different cells [62]. Besides, ROS production by reverse electron transport, RET, in complex I was found to decrease by rotenone as well [63–66]. 

It seems important to say that findings obtained with Cd^2+^ as a probe correlate well with data existed in literature when MPT and/or cell death are produced by different other prooxidants and effectors [48, 67–72]. In particular, Armstrong and colleagues showed that ROS produced by complex III were functionally linked to the MPT pore and the MPT pore opening was critical event leading to death of GSH-depleted leukemic CEM and HL60 cells [48, 68] as well as of human B lymphoma (PW) cells [72]. Besides, there is a lot of other evidence in support of mitochondrial respiration and complex III participation in MPT pore opening and cell death regulation, including data obtained with respiration-deficient clones [73–77]. Furthermore, there are enough of observations in support of complex I involvement in MPT and cell death [78–84], including that on PC12 cells [36]. In particular, Toninello and colleagues showed that salicylate interacted with RLM respiratory chain and produced increase of ROS, which in turn oxidized thiol groups and GSH that in the presence of Ca^2+^ led to the MPT induction; moreover, they found that the reactive group of salicylate for inducing oxidative stress was the hydroxyl group which, by interacting with Fe-S cluster N_2_ of complex I, produced the ROS increase [84]. It is interesting to note that, as considered by several authors, the major site of superoxide generation of complex I is not flavin, but protein-associated ubisemiquinones which are spin coupled with the Fe-S cluster N_2_ [85]. The important data were obtained by several groups of investigators under study of mechanism(s) of action of arachidonic acid [86], ceramides [87–90], and tumor necrosis factor-alpha [91, 92] during induction by them of MPT and apoptosis that also indicated the direct participation of complex I and/or complex III in the processes. In addition, data concerning the contribution of the respiratory chain components in ischemia/reperfusion (hypoxia/reoxygenation) injury [93–100] and the modern knowledge about respiratory supercomplexes [101, 102] including findings on the structure of mitochondrial supercomplex formed by complexes I and III [103–107] are also very impressive.

**Figure 4:**
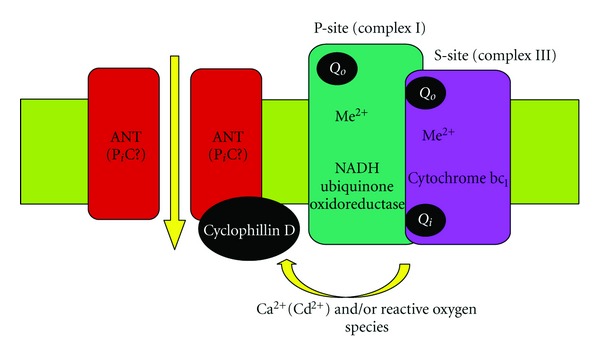
A hypothetical model of mitochondrial permeability transition pore structure: an involvement of a mitochondrial super-complex formed by respiratory chain complexes I and III (modified from [48, 49], see explanation in text).

In general, all the above-mentioned data support our hypothesis about the possible involvement of the mtETC components, namely, a supercomplex formed by complex I (P-site) and complex III (S-site) in the mitochondrial membrane permeabilization mediated by the MPT pore (Figure 4, [16–18, 49]). The hypothesis was suggested by us sufficiently long ago [16] when we for the first time speculated that both respiratory complexes I and III might be involved in the mitochondrial membrane permeabilization promoted by Cd^2+^ and/or Ca^2+^ *plus* P_i_. On the basis of own findings [15, 53, 108] and data existing in literature in the field at that moment, we postulated a hypothetical model of regulated MPT pore. In particular, a model of conventional Ca^2+^-activated CsA-sensitive pore originally proposed by Halestrap (for reviews see [10, 14]) in which ANT was considered its crucial core element (in the light of the modern findings it may be also phosphate carrier, P_i_C) had been integrated with an idea of Fontaine and Bernardi (for review see [9]) concerning mitochondrial respiratory chain complex I involvement in the MPT pore formation and/or regulation. The model had been supplemented by several main postulates, the most important among them were the following. Both complex I and III of the mtETC are places of localization of Ca^2+^(Me^2+^)-binding site(s), critical for the MPT induction, and depending on conditions and cell type, either one or both complexes could be involved in triggering of the MPT pore assembly; besides, the complex I of the respiratory chain likely constitutes the P-site while complex III—the S-site of the MPT pore [16]. Later, the model was extended by additional suppositions [17]. In particular, the complex I (P-site) and complex III (S-site) may constitute not only critical Me^2+^-binding sites but also main loci for ROS generation that was instrumental in oxidation of critical thiol groups and the MPT pore opening [17]. The aforementioned Me^2+^-binding site(s) are most likely disposed: (i) on the way of reverse electron transfer from succinate to NAD+ (complex I); (ii) on cytochrome b somewhere near heme b_L_ and close to stigmatellin binding site (complex III) [17]. Our recent results [18, 109] extended further this theory and indicated that just complex III could be the critical external dithiol [12]. Moreover, it seems that the supercomplex I-III could be the key component of the regulated (i.e., Ca^2+^-dependent CsA-sensitive) MPT pore complex, while complex III is likely involved in the “unregulated” (i.e., Ca^2+^- and/or CsA-insensitive) MPT pore assembly and might concern to the external (inhibitory) Ca^2+^-binding site. Some of these views become more valid in the light of new interesting experimental data obtained by different investigators during the latest time [109–120]; the comprehensive review of the modern ideas and findings on the issue will be given elsewhere (Belyaeva, in preparation). In summary, it is worthy to note that the question of the participation of mitochondrial respiratory chain components in MPT pore formation and/or regulation as well as localization of the critical Me^2+^ binding sites and dithiols is so mysterious and intrigues problems, the decision of which, we hope, could bring new understanding of processes of aging and death.

## 5. Conclusion

In conclusion, the results obtained in this study give new important information to understand the mechanism(s) underlying the heavy-metal-produced neurotoxic action, pointing to the critical involvement of mitochondrial dysfunction in the heavy-metal-induced cell death. We have shown that against the Cd^2+^-induced injury, not only well-known antioxidants and the MPT pore and MCU inhibitors are effective but also different mtETC effectors. Among mtETC effectors under test, only stigmatellin produced the significant and sustained protection of PC12 cells. Moreover, stigmatellin was found to be one of the strongest protectors that exhibited its action not only on different types of the cells (PC12 and AS-30D [6]) but on isolated mitochondria as well [18, 49]. All this points to the direct involvement of the mtETC in the Cd^2+^-induced mitochondrial membrane permeabilization and cell death. In the case of Hg^2+^ and Cu^2+^, all mtETC effectors under study did not exhibit significant protective action on PC12 cells. We concluded that the mtETC dysfunction was involved but in a different way in mechanism(s) of neurotoxic action of the heavy metals. Notably, the data obtained herein further support our hypothesis about the direct involvement of the mtETC components in the mitochondrial membrane permeabilization mediated by the MPT pore assembly [16–18, 49].

## Figures and Tables

**Table 1:** Basal respiration rates (%) of PC12 cells after different time of incubation with increasing concentration of Hg^2+^, Cd^2+^, or Cu^2+^.

Time (h)	Control	[Hg^2+^], *μ*M		[Cd^2+^], *μ*M				[Cu^2+^], *μ*M			
		10	50	10	50	100	500	10	50	100	500
3	45 ± 4	26 ± 5*	3 ± 3*	41 ± 7	25 ± 3*	16 ± 4*	2 ± 2*	43 ± 7	35 ± 7	35 ± 5	35 ± 5
5	35 ± 6					6 ± 3*					
24	39 ± 7	30 ± 2	2 ± 2*	32 ± 2	13 ± 6*	7 ± 1*	0	33 ± 5	32 ± 2	34 ± 5	22 ± 8*

48	41 ± 5	74 ± 26*									15 ± 3*

Rate of oxygen uptake is presented in percentage of the maximal (fully uncoupled) respiration of the control cells. Mean ± SE; *n* = 3 independent experiments; *P < 0.05 compared to untreated control.

**Table 2:** Resting respiration rates (%) of PC12 cells after different time of incubation with increasing concentration of Hg^2+^, Cd^2+^, or Cu^2+^.

Time (h)	Control	[Hg^2+^], *μ*M		[Cd^2+^], *μ*M				[Cu^2+^], *μ*M			
		10	50	10	50	100	500	10	50	100	500
3	13 ± 3	9 ± 1	3 ± 3*	12 ± 3	10 ± 1	5 ± 1*	1 ± 1*	15 ± 3	12 ± 2	14 ± 3	14 ± 1
5	17 ± 2					4 ± 3*					
24	14 ± 2	12 ± 3	2 ± 2*	10 ± 2	5 ± 1*	4 ± 2*	0*	12 ± 1	16 ± 2	13 ± 1	9 ± 3

48	21 ± 2	49 ± 17*									12 ± 2*

Rate of oxygen uptake is presented in percentage of the maximal (fully uncoupled) respiration of the control cells. Mean ± SE; *n* = 3 independent experiments; *P < 0.05 compared to untreated control.

**Table 3:** Uncoupled respiration rates (%) of PC12 cells after different time of incubation with increasing concentration of Hg^2+^, Cd^2+^, or Cu^2+^.

Time (h)	[Hg^2+^], *μ*M		[Cd^2+^], *μ*M				[Cu^2+^], *μ*M			
	10	50	10	50	100	500	10	50	100
3	66 ± 11*	15 ± 3*	77 ± 2*	48 ± 3*	35 ± 3*	4 ± 4*	85 ± 20	81 ± 14	83 ± 14	77 ± 16
5					21 ± 3*					
24	86 ± 7	2 ± 2*	91 ± 13	14 ± 5*	5 ± 2*	0	94 ± 8	82 ± 7	72 ± 6*	58 ± 17*

48	105 ± 5									48 ± 5*

Rate of oxygen uptake is presented in percentage of the maximal (fully uncoupled) respiration of the control cells. Mean ± SE; *n* = 3 independent experiments; *P < 0.05 compared to untreated control.

**Table 4:** Action of different respiratory chain effectors on viability of PC12 cell assessed by LDH release after different time of incubation with different concentration of Cd^2+^.

Treatment (1 *μ*M)	Control cells (3, 5, 24 h)	500 *μ*M Cd^2+^ (3 h)	100 *μ*M Cd^2+^ (5 h)	100 *μ*M Cd^2+^ (24 h)
Control	100	55 ± 3*	74 ± 4*	61 ± 5*
FCCP	96 ± 2	62 ± 10	89 ± 4**	75 ± 10
Rotenone	94 ± 1	59 ± 10	84 ± 8	73 ± 7
Myxothiazol	95 ± 1	52 ± 16	85 ± 7	71 ± 5
Antimycin A	96 ± 2	48 ± 14	82 ± 7	72 ± 6
Stigmatellin	99 ± 2	78 ± 5**	90 ± 5**	76 ± 1**

The cell viability is expressed in % to untreated control. Mean ± SE; *n* = 3–11 independent experiments; *P < 0.05 compared to untreated control; **P < 0.05 compared to Cd(II)-treated control.

**Table 5:** Action of different antioxidants and MPT pore inhibitors on viability of PC12 cells assessed by LDH release after different time of incubation with different concentration of Cd^2+^.

Treatment	Control cells (3, 5, 24 h)	500 *μ*M Cd (3 h)	100 *μ*M Cd (5 h)	100 *μ*M Cd (24 h)
Control	100	55 ± 3*	74 ± 4*	61 ± 5*
Ruthenium Red (10 *μ*M)	99 ± 3	71 ± 6**	92 ± 3**	60 ± 4
BKA, 5 *μ*M	100 ± 2	70 ± 5**	82 ± 6	58 ± 2
CsA, 1 *μ*M	101 ± 2	76 ± 4**	72 ± 6	59 ± 18
NAC (10 mM, 2 h)	104 ± 4	94 ± 2**	96 ± 2**	92 ± 5**
Vit E, 500 *μ*M	103 ± 1	65 ± 6**	86 ± 7**	83 ± 2**
Mannitol (50 mM)	100 ± 1		92 ± 6**	
BHT, 150 *μ*M	99 ± 2		93 ± 1**	82 ± 1**
TEMPO (1 mM)	97 ± 3		87 ± 8**	

The cell viability is expressed in % to untreated control. Mean ± SE; *n* = 3–11 independent experiments; *P < 0.05 compared to untreated control; **P < 0.05 compared to Cd(II)-treated control.

## Abbreviations

mtETC: Mitochondrial electron transport chain
ROS: Reactive oxygen species
MPT: Mitochondrial permeability transition
CsA: Cyclosporin A
CyP-D: Cyclophilin-D
DMEM: Dulbecco/Vogt modified Eagle's minimal essential medium
LDH: Lactate dehydrogenase
ΔΨ_mito_: Mitochondrial membrane potential
FCCP: Carbonyl cyanide p-trifluoromethoxyphenylhydrazone
RCR: Respiratory control ratio
DCFH_2_-DA: Dichlorodihydrofluorescein diacetate
TMRM: Tetramethylrhodamine methyl ester
PBS: Phosphate buffered saline
NAC: N-acetylcysteine
vit E: Vitamin E
BHT: Butylhydroxytoluene
TEMPO: Tetramethylpiperidine-1-oxyl
RR: Ruthenium Red
MCU: Mitochondrial Ca^2+^ uniporter
BKA: Bongkrekic acid
ANT: Adenine nucleotide translocase
P_i_C: Phosphate carrier
PN: Pyridine nucleotides
ISP: Iron-sulfur protein
NEM: *N-*ethylmaleimide
DTT: Dithiothreitol
RLM: Rat liver mitochondria
RET: Reverse electron transport.
